# System Modeling of a MEMS Vibratory Gyroscope and Integration to Circuit Simulation

**DOI:** 10.3390/s17112663

**Published:** 2017-11-18

**Authors:** Hyukjin J. Kwon, Seyeong Seok, Geunbae Lim

**Affiliations:** Department of Mechanical Engineering, Pohang University of Science and Technology, 77 Cheongam-Ro, Nam-Gu, Pohang 37673, Korea; elkei@postech.ac.kr (H.J.K.); athelas@postech.ac.kr (S.S.)

**Keywords:** MEMS, gyroscope, simulation, COMSOL, SPICE

## Abstract

Recently, consumer applications have dramatically created the demand for low-cost and compact gyroscopes. Therefore, on the basis of microelectromechanical systems (MEMS) technology, many gyroscopes have been developed and successfully commercialized. A MEMS gyroscope consists of a MEMS device and an electrical circuit for self-oscillation and angular-rate detection. Since the MEMS device and circuit are interactively related, the entire system should be analyzed together to design or test the gyroscope. In this study, a MEMS vibratory gyroscope is analyzed based on the system dynamic modeling; thus, it can be mathematically expressed and integrated into a circuit simulator. A behavioral simulation of the entire system was conducted to prove the self-oscillation and angular-rate detection and to determine the circuit parameters to be optimized. From the simulation, the operating characteristic according to the vacuum pressure and scale factor was obtained, which indicated similar trends compared with those of the experimental results. The simulation method presented in this paper can be generalized to a wide range of MEMS devices.

## 1. Introduction

Microelectromechanical systems (MEMS) vibratory gyroscopes are among the most commercialized MEMS products. The vibrating structure based on MEMS, instead of complex optical components, allows gyroscopes to be simple, small, and inexpensive compared to conventional optical gyroscopes [1,2,3]. Recently, with the advantages over conventional macro-scale gyroscope, they have been increasingly mass-produced and used in applications ranging from consumer electronics to vehicle. In the case of consumer electronics, they allow gesture recognition in smart phones and image stabilization in cameras. In the automotive case, they are used for vehicle stability control, roll-over detection, and Global Positioning System assistance [4]. A MEMS vibratory gyroscope can be decomposed into a MEMS device and electrical circuit components. The MEMS device includes mechanical transduction of the angular rate into an inertia force via the Coriolis effect followed by electromechanical transduction using a variable capacitor to obtain an electrical signal [5,6]. The electrical circuit component is required for subsequent signal processing and feedback control to ensure that oscillation remains stable. Owing to the integration of silicon MEMS/complimentary metal–oxide–semiconductor, a MEMS device and its electrical circuit components coexist on a small single silicon substrate with low cost and volume [7]. 

In the gyroscope research or development process, simulation is one of the powerful tools that determine the design parameters and characteristic analysis [8]. To simulate the MEMS device components, the MEMS structure and dynamics are usually analyzed using the lumped-parameter model [9,10,11,12,13]. However, a micromachined silicon structure simultaneously acts as both a proof mass and capacitive-sensing electrode. Thus, analyzing both characteristics of the structure together as a system model is important and necessary for efficient optimization. Therefore, equivalent circuit models have been suggested to represent the dynamics of MEMS devices [14,15,16,17] and are integrated into commercial software, namely, MATLAB Simulink (Natick, MA, USA) [18,19]. To consider the thermal or other environmental factors, the finite-element model of a MEMS device is often used, which is characterized by its dynamic flexibility [20,21]. Simulation of the electrical circuit component is relatively simple. Therefore, a commercial circuit simulator, such as SPICE (Cadence, San Jose, CA, USA), is usually utilized to predict the circuit behavior.

Although the devices and circuits are usually verified by numerical simulation, iterations of some design verifications are usually conducted during the prototype design, resulting in an iterative fabrication process. Moreover, the separate designs of MEMS device and circuit components usually lead to system failure or inability to meet performance specifications because the MEMS device and circuit interactively work together [8,22]. Therefore, a significant need exists to simulate both the MEMS device and electric circuit as a complete system for fast and efficient development. The work described in this paper presents a method for system modeling of a MEMS vibratory gyroscope and its integration into an electrical circuit to simulate the entire system.

## 2. Methods

### 2.1. Mechanism of the MEMS Vibratory Gyroscope

**Figure 1:**
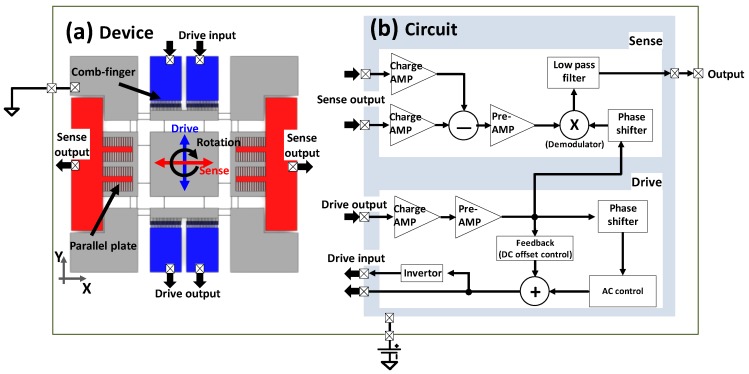
Package of the MEMS vibratory gyroscope system consisting of (**a**) a MEMS device and (**b**) an electrical circuit for self-oscillation at its natural frequency and for output-signal processing.

In this paper, a one-axis MEMS vibratory gyroscope was used for the integrated simulation. This MEMS gyroscope has a high aspect ratio structure, resulting in large capacitance and, thus, high-sensitivity, despite a simple and intuitive structure [23]. The basic concept of this gyroscope is shown at Figure 1a. In this system, a proof mass is suspended by a supporting structure with spring and damper elements. The proof mass is free to oscillate in two principal orthogonal directions: *X* (sense) and *Y* (drive) axes. Through an electrostatic force from the comb finger, the proof mass is driven in an oscillation mode along the *Y*-axis, and this motion is sensed by the change in the capacitance at the comb finger. In response to the *Z*-axis angular rate, the proof mass moves along the *X* (sense)-axis due to the Coriolis force. This motion is sensed by parallel plates at each end of the proof mass, and the amplitude of this motion represents the desired signal proportional to the angular rate. In this study, the signals for electrostatic driving and capacitive sensing are processed by an electric circuit that consists of the driving and sensing parts, as shown in Figure 1b. The drive-axis motion can be set to oscillate at its natural frequency by a simple mechanism that uses an amplifier to compensate for the losses during the vibration. In this research, a self-oscillation mechanism is adapted for best performance. In the drive circuit, the drive output signal created by the drive motion is detected and amplified by a charging amplifier. Then, it is again processed as a drive input signal. To maintain a stable amplitude of the drive oscillation, the DC voltage offset is determined by a feedback system. The sensing circuit basically amplifies the sensed output signal and extracts the angular rate of the drive output using a demodulator.

### 2.2. Gyroscope Dynamics Expressed by an Equation

**Figure 2:**
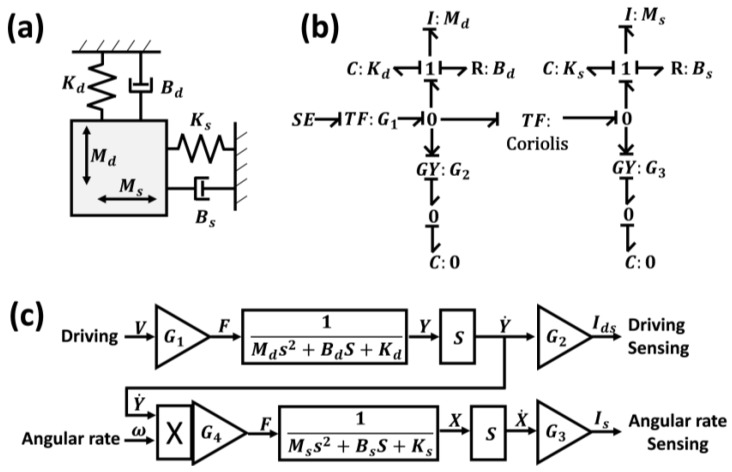
(**a**) Lumped mass–spring–damper model of a gyroscope. (**b**) Bond graph. (**c**) Equivalent system model.

For integration to the circuit simulation, the gyroscope should be expressed as a transfer function. Therefore, the gyroscope was systemically analyzed, as shown in Figure 2a. We assumed that the gyroscope has two degrees of freedom with mass (*M*), spring (*K*), and damper (*B*) elements in the *X* (sense) and *Y* (drive)-axes [24]. This lumped mass–spring–damper model can be expressed as a bond graph, which is a graphical representation of a physical dynamic system, for better understanding of the mathematical relationship [25], as shown in Figure 2b. The effort source (SE), which is the applied input voltage generated by the electrical circuit, was converted into a force on the *Y* (drive)-axis by a transformer (TF:*G*_1_: comb-drive). Consequently, the force induced a motion along the *Y*-axis, which is governed by the mass (*M_d_*)–spring (*K_d_*)–damper (*B_d_*) elements. The velocity of the motion along the *Y*-axis was multiplied by the angular rate of the transformer (TF: Coriolis), thereby becoming a force on the *X* (sense)-axis, which is also governed by the mass (*M_s_*)–spring (*K_s_*)–damper (*B_s_*) elements. The motion in the *Y* and *X*-axis was detected as an electric current through each gyration, namely, GY:*G*_2_: comb sense and GY:*G*_3_: parallel plate sense, respectively. Subsequently, according to the understanding of the dynamics and response characteristics revealed in the bond graph, the equivalent system model of the gyroscope could be described [26], as shown in Figure 2c. Thus, the relationship between the input and output of the gyroscope can be mathematically expressed as follows:

(1) $$\frac{I_{\mathrm{ds}}}{V}=\frac{\left(G_{1}G_{2}\right)s}{M_{d}s^{2}+B_{d}s+K_{d}},$$

(2) $$\frac{I_{s}}{\omega }=\frac{\left(G_{1}G_{3}G_{4}\right)s^{2}}{M_{d}M_{s}s^{4}+\left(M_{d}B_{s}+M_{s}B_{d}\right)s^{3}+\left(M_{d}K_{s}+M_{s}K_{d}+B_{d}B_{s}\right)s^{2}+\left(B_{d}K_{s}+B_{s}K_{d}\right)s+K_{d}K_{s}},$$

where *V* is the voltage input to the gyroscope, *I_ds_* is the *Y* (drive)-axis motion, and *I_s_* is the *X* (sense)-axis motion as a current output from the gyroscope. To complete this transfer function, the mass, spring, and damper elements, namely, *G*_1_*–G*_4_, must also be determined.

### 2.3. Determination of the Mass–Spring–Damper Elements

**Figure 3:**
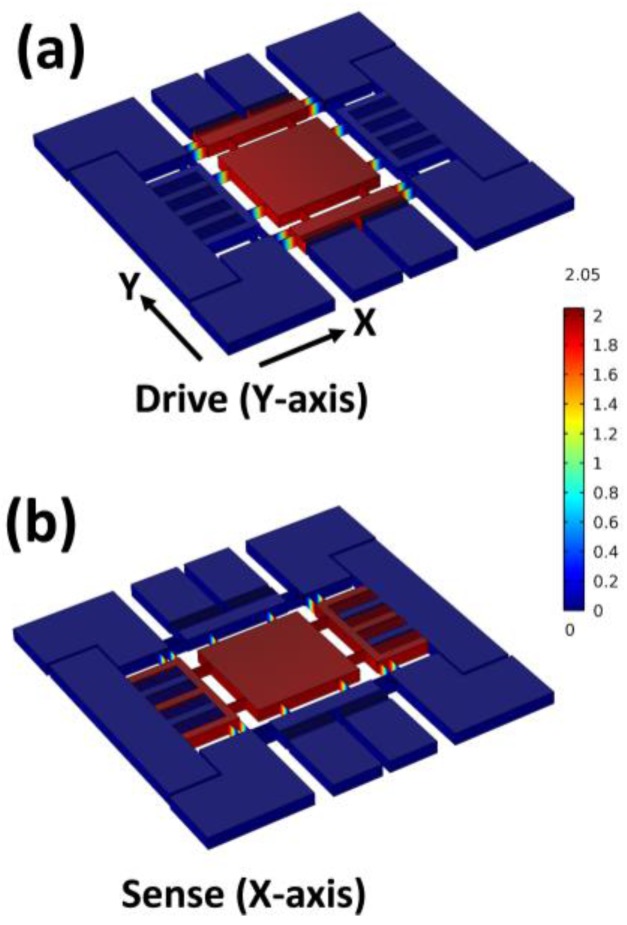
Graphical result of the numerical simulation for the displacement due to the applied force on the (**a**) *Y* (drive) and (**b**) *X* (sense)-axes.

**Figure 4:**
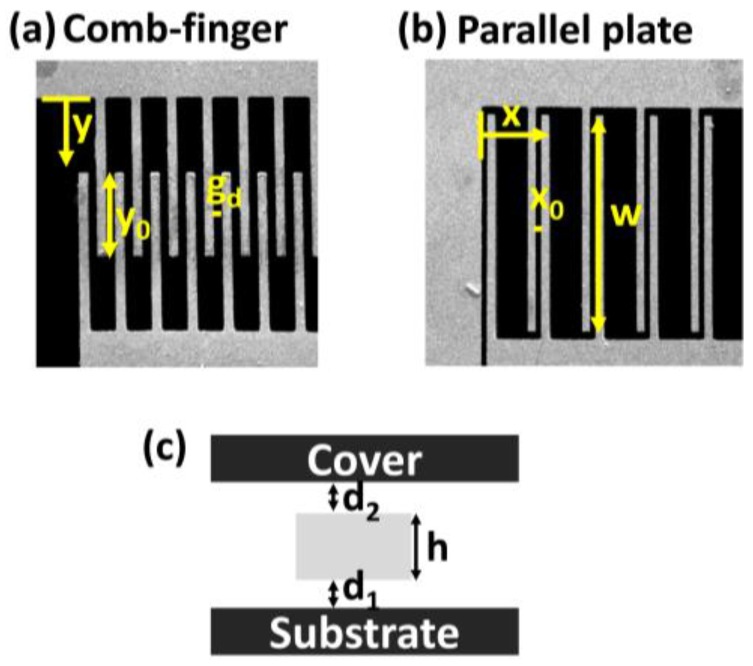
Dimensions of the gyroscope. (**a**) Part of the comb finger: y shows the position along the *Y*-axis, *y*_0_ represents the overlapped length of the electrodes, and g_d_ is the gap between electrodes. (**b**) Part of the parallel plate: *x* shows the position along the *X*-axis, *x*_0_ represents the gap between electrodes, and *w* represents the overlapped length of the electrodes. (**c**) Cross-sectional view: *h* represents the device thickness and *d*_1_ and *d*_2_ are the gaps between the device and substrate or cover.

The mass element can be simply calculated from the product of the area and thickness. To analyze the coupling and spring elements, numerical simulation (COMSOL) was performed, as shown in Figure 3. The applied force on the proof mass was swept to observe the displacement in both axes; thus, the spring constant can be obtained. The coupling between the *X* and *Y*-axes was 0.046%, i.e., they were almost decoupled. Viscous air damping is the dominant damping mechanism in a MEMS gyroscope. To determine the damping value, some variables have been proposed to determine the gyroscope dimension, as shown in Figure 4. The viscous air damping mainly consisted of two elements: (1) slide-film damping, which occurs when two plates slide parallel to each other [27,28], and (2) squeeze-film damping, which occurs when two plates move toward each other [29]. For the drive-axis, slide-film damping occurred between the electrode plates of the comb finger and both at the top and bottom of the suspended proof mass. These relationships can be expressed as follows:

(3) $$B_{comb-finger}=\frac{\mu A_{df}n_{d}}{g_{d}},$$

(4) $$B_{drive\;bottom}=\frac{\mu A_{d}}{d_{1}},$$

(5) $$B_{drive\;upper}=\frac{\mu A_{d}}{d_{2}},$$

where $A_{df}$ is the area of a single plate of the comb finger, $n_{d}$ is number of comb fingers, and $A_{d}$ is the area of the proof mass of the drive-axis. For the sense-axis, slide damping also occurred both at the top and bottom of the suspended proof mass. However, squeeze-film damping occurred between the electrode plates of the parallel plates. These relationships can be expressed as follows:

(6) $$B_{parallel-plate}=\frac{\mu wh^{3}n_{s}}{x_{0}{}^{3}}\beta \left(\eta \right),$$

(7) $$B_{sense\;bottom}=\frac{\mu A_{s}}{d_{1}},$$

(8) $$B_{sense\;upper}=\frac{\mu A_{s}}{d_{2}},$$

where $n_{s}$ is number of parallel plates and $A_{s}$ is the area of the proof mass of the sense-axis. 

### 2.4. Determination of G_1_–G_4_

*G*_1_ represents the relationship between the driving input voltage and electrostatic actuation at the comb finger. Electrostatic actuation relies on the electrostatic attractive forces on the plates with opposite polarities. Thus, the electrostatic force is expressed as follows:

(9) $$F_{electrostatic}=\frac{1}{2}V^{2}\frac{dC}{dy},$$

where *V* is the input voltage and *C* is the comb-drive capacitance. The input voltage can be expressed as follows:

(10) $$V=V_{dc}+V_{ac}\left(t\right),$$

(11) $$V^{2}=2V_{dc}V_{ac}\left(t\right),$$

where *V_dc_* is the DC offset voltage and *V_ac_* is the AC voltage. The above equations show that the resulting electrostatic force depends not only on the applied AC voltage, but also on the DC voltage. The capacitance of the comb drive can be expressed as follows:

(12) $$C_{comb-finger}=\frac{\epsilon_{0}\epsilon_{r}n_{d}\left(y_{0}-y\left(t\right)\right)h}{g_{d}},$$

where $\epsilon_{0}$ is the permittivity of free space and $\epsilon_{r}$ is the relative permittivity of the dielectric. From Equations (9)–(12), *G*_1_ can be finally expressed as follows:

(13) $$G_{1}=\frac{\epsilon_{0}\epsilon_{r}n_{g}hV}{2g_{d}}.$$

*G*_2_ represents the relationship between the velocity in the *Y*-axis motion and the electric current at the comb finger. Capacitive sensors use the current generated by the change in the capacitance for measurements. Thus, under applied voltage $V_{off}$, the current can be expressed as follows:

(14) $$i\left(t\right)=\frac{dQ}{dt}=\frac{dC}{dt}V_{off},$$

where *Q* is amount of charge stored in each plate. From Equations (12) and (14), *G*_2_ can be finally expressed as follows:

(15) $$G_{2}=V_{off}\frac{\epsilon_{0}\epsilon_{r}n_{d}h}{g_{d}}.$$

Capacitive sensing usually requires a high-resolution circuit because the capacitance in a MEMS device is normally very small. *G*_3_ represents the relationship between the velocity in the *X*-axis motion and the electric current in the parallel plate. From Taylor’s expansion, the capacitance of the parallel plate can be expressed as follows:

(16) $$C_{parallel-plate}=\frac{\epsilon_{0}\epsilon_{r}n_{s}wh}{x_{0}+x\left(t\right)}=\frac{\epsilon_{0}\epsilon_{r}n_{s}wh}{x_{0}}-\frac{\epsilon_{0}\epsilon_{r}n_{s}wh}{x_{0}{}^{2}}x\left(t\right).$$

From Equations (14) and (16), *G*_3_ can be finally expressed as follows:

(17) $$G_{3}=V_{off}\frac{\epsilon_{0}\epsilon_{r}n_{s}dh}{x_{0}{}^{2}}.$$

Finally, *G*_4_ represents the relationship between the angular rate and Coriolis force applied on the *Y*-axis. The Coriolis force is expressed as:

(18) $$F_{Coriolis}=2m_{s}\omega \dot{y}\left(t\right),$$

where Ω is the angular rate. The Coriolis force also depends on the motion in the *Y*-axis; thus, the amplitude of the drive oscillation should be well maintained to achieve a stable scale factor. *G*_4_ can finally be expressed as follows:

(19) $$G_{4}=2m_{s}.$$

### 2.5. Integration of the Gyroscope and Electrical Circuit

**Figure 5:**
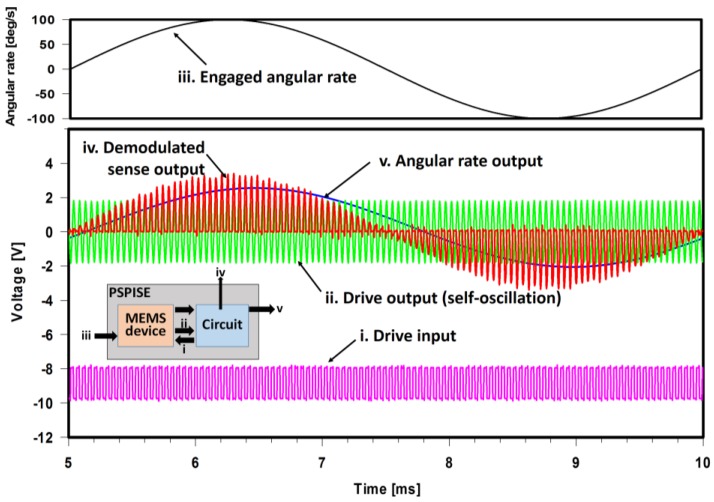
Signals from the combined simulation of the gyroscope and its driving and sensing circuit. (i) Drive input: the signal generated from circuit and applied on electrodes at MEMS device for oscillation. (ii) Drive output: the oscillation signal detected by electrodes at MEMS device followed by an amplification. (iii) The rotation engaged to the gyroscope. (iv) The demodulated sensing signal containing the applied angular rate. (v) The extracted angular rate output.

From the determination of the mass, spring, and damper elements in each axis and the *G*_1_*–G*_4_ values, the MEMS device can be converted into a complete transfer function using Equations (1) and (2). Thus, it was implemented in the circuit simulation (PSPICE) to analyze the entire system consisting of the MEMS device and electrical circuit. In the simulation, force and angular rate applied on the MEMS device are converted to a voltage signal for the process. Figure 5 shows the signals from the combined simulation which indicates that the gyroscope and the electrical circuit were completely simulated. In the circuit, the drive signal (i) was composed of the AC signal from the drive output signal (ii) itself for self-oscillation and the DC offset generated by the feedback circuit to maintain the oscillation amplitude. Since the motion of the moving electrodes induces a capacitance change, the signal of the drive and sense output from the MEMS device are actually produced in current form (Note that signal as drive output (ii) was measured right after the pre-amp). The drive output signal shows that the gyroscope was self-oscillating. In response to the applied angular rate (represented by voltage signal in simulation) (iii) applied on the gyroscope, the sensing signal was demodulated (iv) and, thus, became the output of the gyroscope (v) through a low-pass filter. 

### 2.6. Dimension and Experimental Setup of Actual Gyroscope Device

In order to verify the integrated simulation, an actual MEMS device having certain parameters (Table 1) and an electric circuit was fabricated. Low-resistivity silicon (300 µm) and Pyrex glass (500 µm) wafers are used to fabricate the gyroscope by using a bulk micromachining process. First, the front side of the silicon wafer was etched and patterned by deep ion reactive etching (DRIE) to obtain a high aspect ratio structure. Then, wet etchant was used to etch the backside of the silicon wafer where the moving parts are supposed to be suspended from substrate, such as the proof mass and comb finger. Finally, the unetched surface (anchor) on the silicon backside and glass (substrate) are bonded by an anodic bonding technique. To operate and test the fabricated MEMS device, it was interfaced to an electric circuit on a printed circuit board (PCB) and placed on the rate table in a vacuum chamber instead of vacuum encapsulation to control the pressure (101.35 to 0.13 Pa). When the angular rate was engaged by the rate table, the output voltage as the output angular rate was recorded by LabVIEW through data acquisition equipment (DAQ-9172, NI, Austin, TX, USA) and the various signals were captured by an oscilloscope (DL1740, Yokogawa, Tokyo, Japan) and thus compared with the simulation result.

## 3. Result

### 3.1. Natural Frequency of the Drive and Sense Axes

**Figure 6:**
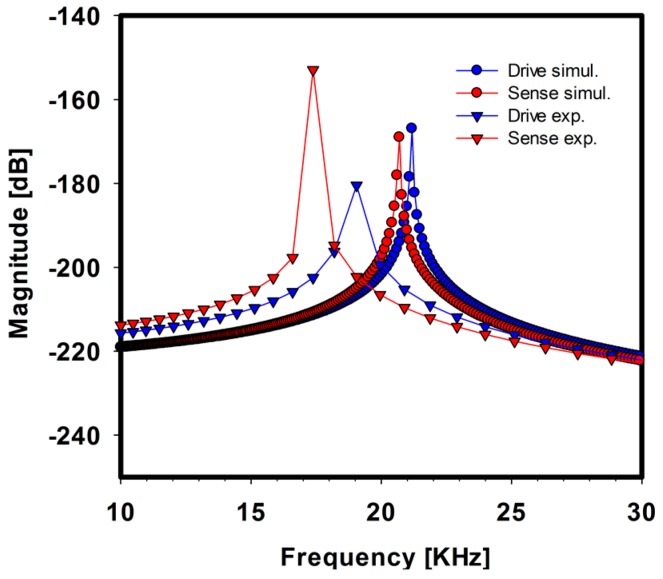
Comparison between the simulation and experimental results of the frequency sweep showing the natural frequency of each drive and sense-axis.

According to the system modeling of the gyroscope, the frequency response was obtained by simulation. For comparison, the actual device was also measured, as shown in Figure 6, which indicates its natural frequency. In the simulation, the drive and sense axes had 10% and 16% lower natural frequency, respectively, than the actual measurement. We assumed that the spring element, which is the support structure for the proof mass, was over-etched, thus resulting in a lower spring constant and natural frequency.

### 3.2. Determination of the Q Factor

**Figure 7:**
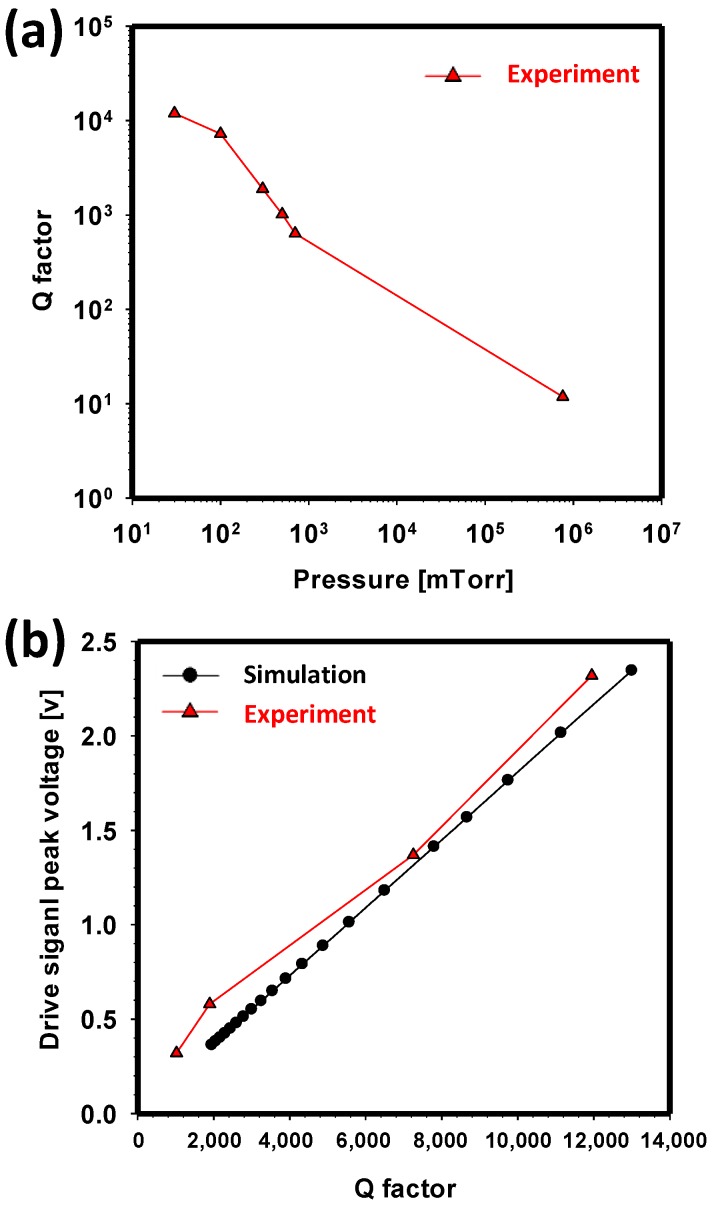
(**a**) Experimental result of the quality factor as a function of air pressure. (**b**) Comparison of the simulation and experimental results of the driving signal peak voltage versus air pressure.

The damper element, which is the friction mainly caused by viscous-film damping, plays an important role in the gyroscope performance, such as the amplitude of the drive and sense oscillations. Thus, maintaining a high quality factor (lower damping coefficient) results in lower power consumption, improved stability, and increased sensitivity. Therefore, a gyroscope is usually vacuum-packaged to minimize viscous damping. Thus, accurate *Q* factor prediction as a function of the vacuum pressure is highly needed. Although the damper element can be estimated using a simplified equation, as mentioned earlier, theoretically estimating it for a complicated gyroscope system is usually very difficult compared with the other factors due to the complexity and nonlinearity of the fluid motion [30]. Hence, the quality factor at various air pressure values is measured using an actual device, as shown in Figure 7a. Then, simulation is conducted based on this parameter. To compare and validate the experiment, a drive circuit without feedback control is connected to the gyroscope for self-oscillation and measurement of its drive signal in both simulation and experiment. Both results show similar trends, as shown in Figure 7b.

### 3.3. Optimization of Demodulation and Minimizing a Quadrature Error

**Figure 8:**
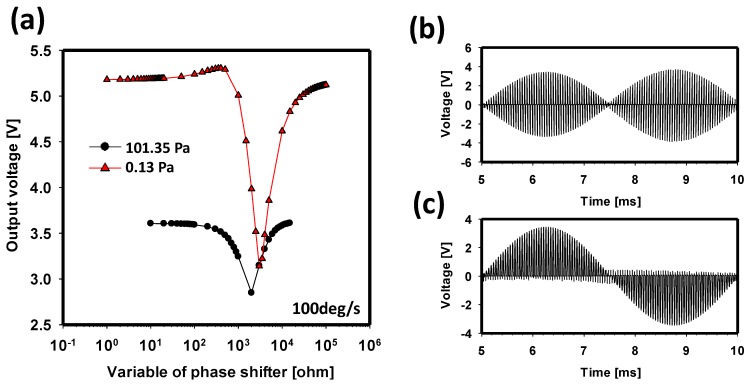
(**a**) Output voltage (angular–rate signal) as a function of the resistance of the phase shifter in response to 100°/s. (**b**) Demodulated signal using a phase shifter with 3 kΩ. (**c**) Demodulated signal using a phase shifter with 560 Ω.

When subjected to rotation around the *Z*-axis, the proof mass vibrates along the *X* (sense)-axis, and the vibration amplitude caused by self-oscillation is modulated by the applied angular rate. This modulated sensing signal is detected by the sensing circuit and is demodulated using the drive signal from the drive circuit. In this process, the phase of the sensing and driving signals is assumed to have been matched to obtain the best sensitivity. Therefore, a phase shifter is required to adjust the phase of the drive signal, which means that it is one of the most critical parts of the test circuit in the gyroscope. We demonstrate the optimization of the phase shifter at the demodulator using integrated simulation. Figure 8a shows the output voltage as a function of the phase-shifter parameter under two different pressure values when a constant angular rate is applied. The graph shows that an optimized parameter can be found, and it varies with the air pressure. To compare the results between the optimized and worst values, Figure 8b,c show the demodulated signal by the phase shifter with 3 kΩ and 560 Ω, respectively. At 3 kΩ, the shape of the signal indicates that the angular-rate signal is not fully extracted. In contrast, the phase of the drive signal is shifted in 90° with 560 Ω, thereby demodulating the angular-rate signal well. Thus, the largest output voltage can be obtained, as shown in Figure 8c. Subsequently, the frequency of the carrier signal is eliminated by the low-pass filter to obtain the complete angular-rate signal.

**Figure 9:**
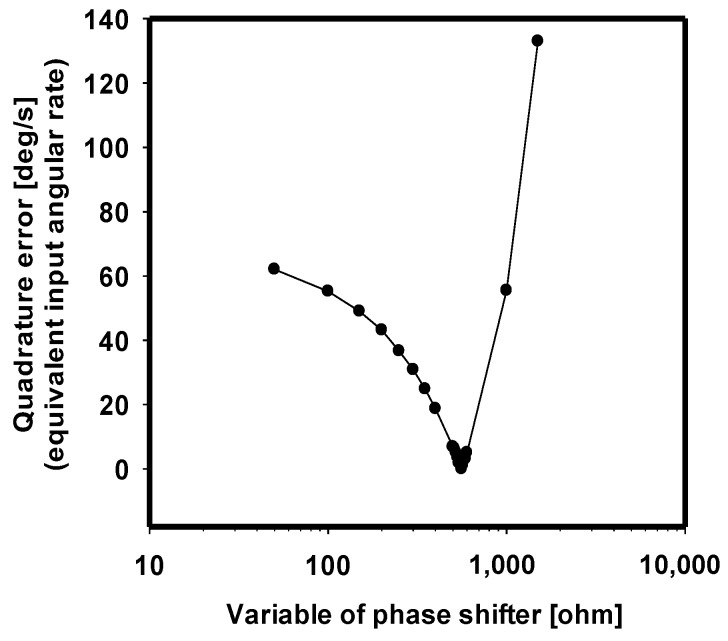
Quadrature error as a function of the resistance of the phase shifter in response to 100°/s. The graph indicates a quadrature error of 0.00323°/s at the significant optimal point, 560 Ω.

The optimization of demodulation is also inevitable to reject quadrature error. Due to mechanical imperfection, a small portion of the drive motion is also delivered directly to the sense-axis. This coupling creates an undesired feedthrough signal known as quadrature error due to its 90° phase shift relative to the drive signal. Generally, the quadrature error is much larger than the signal of the Coriolis Effect and, thus, must be rejected by demodulation. Figure 9 shows the quadrature error as a function of the phase-shifter parameter. The graph shows that quadrature error is almost rejected at the optimal point, 560 Ω, where the sense output signal is demodulated by exactly the same phase as the drive output signal.

### 3.4. Output Voltage as Function of Angular Rate

**Figure 10:**
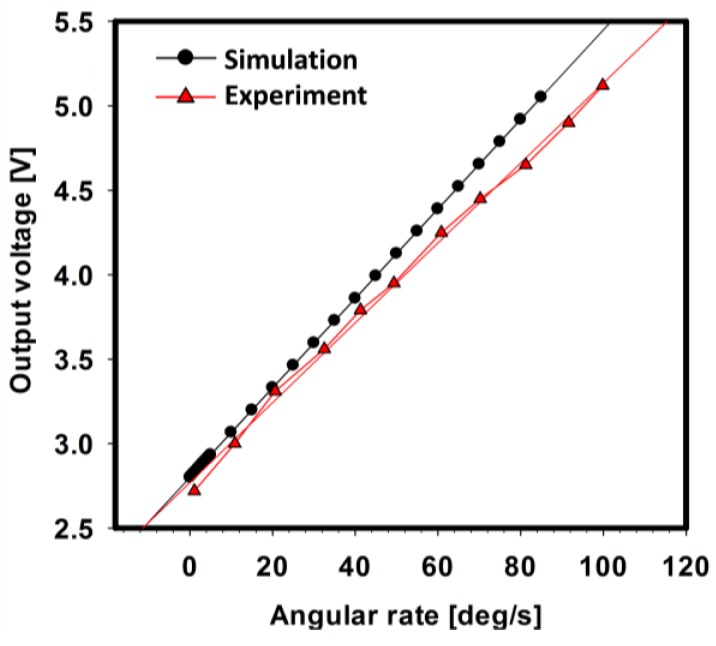
Comparison of the scale factor between the simulation and experiment.

On the basis of the optimization by simulation, a test circuit was fabricated and integrated with an actual gyroscope. Similar to the simulation result, the gyroscope was successfully self-oscillated and responded to an angular rate. Figure 10 shows that the output characteristics of the gyroscope were measured from both simulation and experiment at 30 mTorr. The result shows that the voltage of the gyroscope was linear and a function of the angular velocity with a scale factor of 26.5 mV/(°/s) in the simulation and 23.6 mV/(°/s) in the experiment. The result reveals that this integrated simulation represented the operation of an actual device with an electrical circuit well.

## 4. Discussion

In this study, the system model of a MEMS vibratory gyroscope is developed using a bond graph and was integrated into an electrical circuit for simulation. A behavioral simulation of the entire system, which included both the MEMS device and the electrical circuit, was conducted. Thus, self-oscillation and detection of the angular rate were demonstrated, and we optimized a phase-shifter value at vacuum pressure. Although we assumed that fabrication imperfections of the gyroscope mainly contributed to the spring term, which resulted in a small difference between the experiment and simulation, the result showed a good correlation of the results. Instead of time-consuming laboratory experiments and costly MEMS or circuit fabrication processes, to minimize the cost and time in research and development, the simulation method provided in this study can be generalized to a wide range of MEMS devices, which can help in the design of MEMS devices and in determining the optimized parameters of a test circuit.

## Figures and Tables

**Table 1:** Parameters of the gyroscope.

Parameter	Symbol	Value
Gap between sense electrodes	*x*_0_	9.46 µm
Length of overlapped comb finger	*y*_0_	135 µm
Thickness of the device	*h*	250 µm
Gap between device and substrate	*d*_1_	50 µm
Gap between device and cover	*d*_2_	500 µm
Gap between comb fingers	*g_d_*	14.5 µm
Length of overlapped parallel plates	*w*	2.4 mm
Proof mass area of the drive-axis	*Ad*	7.93 mm^3^
Proof mass area of the sense-axis	*As*	8.48 mm^3^
Area of a single plate of comb finger	*A_df_*	0.0338 mm^3^
Number of comb finger	*n_d_*	164
Number of parallel plate	*n_s_*	112
